# Approach To Treatment-Resistant Recurrent Depressive Disorder

**DOI:** 10.1192/j.eurpsy.2026.11408

**Published:** 2026-07-31

**Authors:** M. J. Muñoz Algar, P. Bernal García

**Affiliations:** Psychiatry, Hospital Dr. Rodríguez Lafora, Madrid, Spain

## Abstract

**Introduction:**

The most accepted definition of treatment-resistant depression (TRD) is the failure of at least two adequate antidepressant treatments during the current episode (Cleare,A.J.,et al.BMC Psychiatry,2019;19,(1)223). Therefore it is necessary to confirm that a correct diagnosis has been made, ensure proper adherence to the treatment, confirm that appropriate doses and durations have been received, and rule out possible comorbidities (such as bipolar disorder, substance abuse, and medical causes) (Fornaro,M.,& Giosuè,P. CPEMH 2010;6,20-24).

**Objectives:**

To analyze the case of a woman with recurrent depressive disorder who presented resistance to several treatment changes, including those with stronger scientific evidence.

**Methods:**

A descriptive study of the case of a 49-year-old woman who was admitted several times due to severe recurrent depressive resistant disorder. She has no relevant medical history, no history of substance abuse but she has several family psychiatric history of affective disorders and suicide.

**Results:**

The patient was admitted due to low mood, anhedonia, thought blocking, feelings of hopelessness, weight loss and insomnia. The SSRI she was taking was discontinued and a dual-action antidepressant was introduced with several changes in hypnotic medication leading to partial recovery and subsequent discharge. One month later, she was readmitted because she experienced a sudden worsening of her depression, with inhibition and suicidal ideation. Duloxetine was increased to the maximum dose with minimal improvement, and electroconvulsive therapy (ECT) was considered and administered, resulting in improved sleep, mood, and reduced anxiety after 11sessions, but without a full recovery.

Three weeks later, she was readmitted in hospital with marked clinical deterioration. The levothyroxine that was started before discharge was discontinued due to lack of efficacy, and valproate was started up to 700 mg/day. The antidepressant was changed to desvenlafaxine with side effects. Given the inefficacy of several previous treatments, intranasal esketamine was initiated (up to 56 mg twice weekly during the induction phase), resulting in a reduction in suicidal ideation and obsessive ruminations. Finally, the dual-action antidepressant was replaced by a tricyclic (clomipramine 225 mg/day), achieving a slow but significant improvement, with restored capacity for enjoyment and calm, disappearance of suicidal thoughts, and development of future plans.

**Conclusions:**

The current treatment guidelines of TRD recommend a stepped strategy: optimization→change→combination→augmentation→intensive psychotherapy→somatic treatments (ECT, transcranial magnetic stimulation (TMS), esketamine). ECT has the strongest evidence in severe major depression; TMS and esketamine are useful in TRD with specific criteria. Finally, psychotherapy combined with pharmacotherapy improves outcomes and should always be considered.

**Disclosure of Interest:**

None Declared

